# Trait Anxiety, Emotion Regulation, and Metacognitive Beliefs: An Observational Study Incorporating Separate Network and Correlation Analyses to Examine Associations with Executive Functions and Academic Achievement

**DOI:** 10.3390/children11010123

**Published:** 2024-01-18

**Authors:** François-Xavier Cécillon, Martial Mermillod, Christophe Leys, Jean-Philippe Lachaux, Sarah Le Vigouroux, Rebecca Shankland

**Affiliations:** 1 Laboratoire Développement Individu Processus Handicap Education, Université Lumière Lyon 2, 5, Avenue Pierre Mendès-France, 69676 Bron, Cedex, France; rebecca.shankland@univ-lyon2.fr; 2 Laboratoire Psychologie et NeuroCognition, CNRS, Université Grenoble Alpes, 38000 Grenoble, France; martial.mermillod@univ-grenoble-alpes.fr; 3 Faculté de Psychologie, Sciences de l’Education et Logopédie, Université Libre de Bruxelles, Avenue Franklin Roosevelt, 50—CP191, 1050 Bruxelles, Belgium; christophe.leys@ulb.be; 4 Centre de Recherche en Neurosciences de Lyon, Bâtiment 452—95 Bd Pinel, 69500 Bron, France; jp.lachaux@inserm.fr; 5 Laboratoire APSY-V, Université de Nîmes, 30021 Nîmes, Cedex 1, France; sarah.le_vigouroux_nicolas@unimes.fr; 6 Institut Universitaire de France, 1 Rue Descartes, 75231 Paris, Cedex 05, France

**Keywords:** trait anxiety, emotion regulation strategies, metacognitive beliefs, academic success, executive functioning, attentional control theory, adolescents

## Abstract

Trait anxiety, emotion regulation strategies, and metacognitive beliefs influence executive functions (EFs) and academic achievement. This study examines their interplay and impact on academic success. In total, 275 adolescents (10–17 years) and parents completed an online questionnaire assessing trait anxiety, emotion regulation strategies, metacognition, parent-reported behaviors related to executive functioning, and overall school average. Preliminary analyses confirmed consistency with the existing literature for each variable and their interaction. Furthermore, we conducted a network analysis among the main variables. This analysis supports the need to pay more attention to reflective variables—maladaptive emotion regulation strategies and metacognitive beliefs about worry—when studying trait anxiety. These variables were linked to problematic executive functioning in adolescents, and the latter was negatively linked to academic achievement. This study offers innovative insights by investigating relationships less explored in the scientific literature. It reveals high and significant correlations between metacognitive beliefs, maladaptive emotion regulation strategies, and trait anxiety (r > 0.500, *p* < 0.001) but also between these variables and both executive functioning and academic achievement. These findings offer new perspectives for research and underscore the importance of holistically examining the psychological factors related to academic success.

## 1. Introduction

For decades, the influence of anxiety on academic performance has been extensively studied. Numerous theories have attempted to explain how anxiety affects performance, but consensus remains elusive. Nonetheless, identifying anxiety’s essential mechanisms is crucial for early prevention and mitigation, especially in education. Studies show that individuals with anxiety disorders are at a higher risk of dropping out of high school and encounter difficulties in obtaining diplomas [1,2]. Anxiety’s negative effects extend beyond clinical populations to subclinical ones in various academic subjects and can impact long-term academic achievement [3,4]. The school environment imposes constraints leading to anxiety, hindering goal achievement. Situational anxiety, a transient state triggered by perceived threats, depends on the situation and predisposition to anxiety [5]. Trait anxiety, a dispositional form, involves intrusive thoughts, worry, difficulty disengaging from negativity, and physiological manifestations [6]. It heightens sensitivity to threats or negative information, impeding cognitive abilities for knowledge integration and retrieval. The relationship between trait anxiety and academic performance is deeply rooted in cognitive and emotional processes. When students engage in tasks like mathematical exercises, they mobilize various cognitive functions such as attention, memory, and reasoning. However, trait anxiety can disrupt these processes. According to Ellis and Ashbrook’s theory of resource allocation [7], emotional states, including anxiety, can interfere with the attentional resources needed for memory tasks. This leads to a state of hypervigilance, as described in the S-REF model by Wells and Matthews [8], where anxiety-driven thoughts compete with task-focused cognitive activities. The attentional bias towards perceived threats, a key feature in anxious individuals, further exacerbates this issue [9]. This bias not only affects the perception of immediate threats but also the processing of complex information, leading to a cognitive overload that hinders academic performance [10]. Thus, trait anxiety can significantly impact a student’s ability to perform academic tasks efficiently by diverting cognitive resources and amplifying threat perception. Trait anxiety could potentially hinder academic success by impacting the key cognitive functions essential for learning, such as executive functions, although this is not always the case [11,12,13]. 

### 1.1. Executive Functions and Academic Achievements

Concerning the link between executive functions (EFs) and academic achievement, the cognitive functions primarily affected by trait anxiety are executive functions (EFs). EFs are responsible for controlling and coordinating specific cognitive processes [14], particularly in learning, to direct students’ behaviors toward specific goals and facilitate the integration of new knowledge. In this regard, the literature has shown that EFs play a crucial role in academic achievement [15,16]. Best et al. (2011) studied the link between complex executive function (EF) tasks, requiring coordination of multiple EF components, and academic performance in 1395 children and adolescents (5–17 years) using the Cognitive Assessment System [12]. They found significant and positive correlations between these tasks and performance in both mathematics and reading, suggesting a broad relationship between complex EFs and academic success. This finding applies to both performance-based and rating-based EF measures. For example, a study by McAuley et al. [17], which utilized the Behavior Rating Inventory of Executive Function (BRIEF) [18], a parent-reported questionnaire assessing problematic behaviors related to executive functioning in children and adolescents in daily life, showed significant negative correlations between EFs and mathematical and reading abilities in a sample of 97 children aged 6 to 15 years. Similar results were later replicated in a larger study [19]. However, these two studies found differences between performance-based and rating-based EF measures.

Concerning performance-based EFs vs. questionnaire-based EFs, although the BRIEF is one of the most commonly used scales having reliable psychometrics for adolescents [20], it, like other questionnaire-based EF measures, has been subject to extensive discussions regarding what they actually assess. Indeed, out of 20 studies that examined the association between performance-based EF measures and rating-based EF measures, only 24% of correlation comparisons were found to be significant, with correlations being relatively weak in magnitude (median *r* = 0.19) [21]. Drawing from Stanovich’s [22,23] framework, different measures of executive functioning can be categorized into two distinct levels: reflective analysis and algorithmic analysis. Reflective analysis, as reflected in rating-based EF measures, involves considering an individual’s goals and beliefs related to those goals. This level of analysis focuses on the conscious reflection and evaluation of one’s own cognitive processes and decision-making strategies. It involves introspection and self-consciousness regarding the alignment of actions with goals and beliefs, leading to the selection of rational actions based on these reflections. On the other hand, algorithmic analysis, as indicated by performance-based EF measures, encompasses the efficiency of information processing mechanisms in the brain. This level of analysis involves cognitive processes such as information encoding, perceptual registration, working memory, and other cognitive abilities. Ultimately, performance-based and rating-based EF measures refer to the same construct but assess different components of that construct [21]. Nevertheless, both measures remain relevant for use in the educational context, as demonstrated by the previously discussed studies.

### 1.2. Executive Functions and Trait Anxiety

Concerning the link between EFs and trait anxiety, the Theory of Processing Efficiency (TPE) [24] and the Attention Control Theory (ACT) [25] posit that trait anxiety impairs the efficiency of at least three core EFs [14] through attentional control: inhibition, shifting, and updating [25,26]. Performance itself may not necessarily be affected, unlike processing efficiency. In other words, the cognitive cost of a task is greater for individuals with high trait anxiety [27]. This distinction is explained by the orientation of attention toward irrelevant stimuli for task completion among anxious individuals and the implementation of compensatory strategies that rebalance performance quality compared to individuals with lower anxiety levels. This postulate clarifies the occasional absence of a negative relationship between anxiety and measured performance, or even the observation of a positive relationship. Advocates of the ACT recently concluded that cognitive neuroscience provides strong evidence for the involvement of several factors in the alteration of processing efficiency associated with high trait anxiety [6]. They propose that global conceptualizations of processing efficiency be abandoned as overly simplistic. According to them, researchers should focus on more specific conceptualizations by distinguishing between relevant and irrelevant processing efficiency in anxious individuals. Non-relevant processing interventions would occur sooner in highly anxious individuals compared to those with low anxiety levels. These interventions would then fade away, giving rise to more task-relevant processing through the use of compensatory strategies aimed at redirecting processing resources toward tasks requiring the utilization of EFs, such as academic learning.

### 1.3. Emotion Regulation Strategies

The process of emotion regulation (ER) could be one of the compensatory mechanisms that redirect resources toward a specific goal. On the one hand, emotions themselves are considered to lead to expressive, goal-oriented, and adaptive behaviors [28]. On the other hand, the general conception of emotion regulation is that “Emotion regulation consists of the extrinsic and intrinsic processes responsible for monitoring, evaluating, and modifying emotional reactions, especially their intensive and temporal features, to accomplish one’s goals” [29]. ER would intervene to reduce emotional interference and allow cognitive resources to be deployed toward the ongoing task. A portion of the literature on emotion regulation has focused on conscious regulation strategies to cope with unpleasant emotions, such as those assessed by the Cognitive Emotion Regulation Questionnaire (CERQ) [30]. Concerning ERSs and anxiety, in a meta-analysis focusing on adolescents aged 13 to 18 [31], significant associations were found between both adaptive and maladaptive emotion regulation strategies (ERSs) and symptoms of anxiety and depression. This is consistent with previous research, such as a study by Aldao et al. [32], which observed similar patterns in adults, linking maladaptive ERSs like rumination, avoidance, and suppression with greater psychopathology. In adolescents, strategies like reappraisal, suppression, problem solving, acceptance, avoidance, and rumination showed varying degrees of effect sizes, indicating their different impacts on anxiety and depression. Both adaptive and maladaptive ERSs were equally important in these disorders. Regarding the CERQ specifically, a Japanese meta-analysis investigating these strategies using the CERQ and their relationship to anxiety (eight studies) and depression (16 studies) confirmed previous findings (blaming others was significantly linked to anxiety and depression but had the smallest absolute value) and highlighted a positive link between acceptance and anxiety and depression [33]. As Garnefski and Kraaij [34] emphasize, drawing on Wilson’s [35] work, acceptance can be applied actively as a form of self-assertion or passively as a form of resignation. Therefore, the questionnaire is sensitive, particularly for this strategy, to how each individual conceptualizes acceptance. Despite the limitations of this tool and the binary conceptualization of ERSs, ERSs could be a promising candidate for explaining the impact of anxiety on executive functions. However, research has shown that emotional dysregulation predicts psychopathology, but the reverse is not true [36], suggesting that ERSs may act upstream of trait anxiety.

Concerning the link between ERSs and EFs, Lantrip et al. [37] linked ERSs and EFs, noting that maladaptive ERSs correlate with poor executive functioning in adolescents, while adaptive ERSs indicate better functioning. However, their study, using self-reported ERQ-CA [38,39] and BRIEF data, had a modest sample (70 adolescents) and limitations. Other research suggests that cognitive abilities, particularly working memory, are crucial for emotion regulation [40,41,42], but the relationship between EFs and ERS development is complex, as shown by mixed results in working memory training [43] and Veloso and Ty’s study [44], which did not find a change in ERSs despite reduced trait anxiety through EF training. Ultimately, while cognitive abilities certainly play a role and facilitate our way of regulating emotions [40,41], the question of why individuals choose maladaptive ERSs over adaptive ones to diminish the impact of emotion on information processing remains unanswered.

### 1.4. Metacognitive Beliefs

Wells [45] suggested that metacognition, representing the knowledge or cognitive activities that regulate and organize mental functioning [46,47], not only allows us to pay attention to what enters our consciousness but also to evaluate and influence the types of strategies we use to regulate thoughts and feelings. It plays a central role in how individuals experience unpleasant emotions and negative self-evaluations. Wells and Matthews developed the Self-Regulatory Executive Function (S-REF) model [8], which posits that similarities in emotional disorders are due to a negative and persevering thinking style called the cognitive attentional syndrome (CAS) [45]. Conceptualized in terms of traits, this thinking pattern is regulated by metacognitive knowledge (i.e., metacognitive beliefs) that is measured by five subcomponents in the reference questionnaire (MCQ-65) [48]: (1) Positive Beliefs about Worry (MCpos), (2) Negative Beliefs about Worry’s Uncontrollability and Danger (MCneg), (3) Beliefs on the Need to Control Thoughts (Control), (4) Beliefs about Cognitive Competence (Lack of Confidence), and (5) Cognitive Self-Consciousness (Consciousness). A recent study reported that this scale could explain 83% of the variance in anxiety propensity in adults [49]. The same study found that negative beliefs contributed significantly to anxiety. However, the researchers emphasized that metacognitions can both predict anxiety and be predicted by anxiety. This bidirectional link highlights the interplay between traits (metacognitive beliefs/anxiety dispositions) and states (metacognitive strategies used/situational anxiety), making it challenging to separate these concepts temporally. However, the theory posits that this thinking style leads to worry, heightened threat monitoring, and the development of maladaptive coping strategies such as rumination, which impairs self-regulation [45]. It is therefore believed to act primarily and to significantly increase trait anxiety. Furthermore, this model suggests that cognitive abilities are not the cause of performance difficulties in individuals with this thinking style; rather, the cause is the individual’s relationship with their own thoughts and the choice of strategies used to regulate their emotions.

### 1.5. Theorical Framework and Hypotheses

This study is primarily correlational and relies on questionnaires filled out by adolescents and a referring parent. Initially, it was important to verify the consistency of our data with the existing literature and to expand the links between EFs and ERSs, as well as between metacognitive beliefs and ERSs. Previous studies indicate that ERSm are often associated with higher levels of anxiety, more so than ERSa. **Hypothesis 1** (posted in OSF): *we anticipated a strong correlation between maladaptive ERSs and trait anxiety and a weaker correlation for adaptive ERSs. Executive functions have strong theoretical and structural links with emotion regulation*. **Hypothesis 2** (posted in OSF): *the use of ERSa was expected to be positively associated with better executive functioning and the use of ERSm to be negatively associated. Indeed, Wells and Matthews’s theory suggests that metacognitive beliefs influence the strategies we use to regulate our emotions*. **Hypothesis 3** (not posted in OSF): *we expected that metacognitive beliefs would influence the choice of ERS by adolescents. This theory also posits that metacognitive beliefs reinforce participants’ trait anxiety*. **Hypothesis 4** (posted in OSF): *we expected to observe a positive influence of metacognitive beliefs on teenagers’ trait anxiety*.

The ACT has thus far considered the relationship between anxiety and EFs at an algorithmic level (treatment effectiveness) rather than at a reflective level (decisions made based on individuals’ beliefs and goals). In this study, we examined the influence of this reflective level through metacognitive beliefs and ERSs. We believe that metacognitive beliefs play a crucial role in trait anxiety through the orientation they provide to the employed ERSs. Consistent with ACT predictions, we postulate that emotion regulation strategies could act as compensatory factors that limit anxiety and its impact on executive functioning. Therefore, we expect that the choice of maladaptive ERSs will explain the deleterious effect of trait anxiety on EFs. Furthermore, we anticipate that adolescents exhibiting problematic behavior, reflecting poor executive functioning, will have lower academic achievement compared to adolescents with more directed and controlled behaviors. **Hypothesis 5** (posted in OSF): *in a comprehensive model, we hypothesized that the impact of trait anxiety on EFs could be mediated by the choice of employed ERSs. This relationship between trait anxiety and ERSs would be moderated by adolescents’ metacognitive beliefs*.

## 2. Materials and Methods

### 2.1. Sample

The sample consisted of 292 adolescents aged 10 to 17 years (M = 12.47; SD = 1.79), including 53.08% girls and 1 “other” gender category. The link to the questionnaire was shared via social networks and sent to schools in Lyon, France, and its immediate suburbs. More precisely, two middle schools in the Lyon suburbs and one in the south of France distributed the questionnaire to parents, which explains the large number of middle-school students in our sample. Regarding educational level, the majority of participants were in middle school (80.82%), followed by high school (15.75%). Only 10 participants (3.42%) were in elementary school. At the time of questionnaire completion, 288 participants reported that the decision made for them the previous year was to progress to the next grade. A different decision was made for 4 participants (3 for reorientation or changing schools, and 1 reporting a “very bad year”). Overall, 86.98% of the sample attended public schools, 11.98% attended private schools, and 1.02% were in completely private institutions (without contracts with the government). The overall mean grade of the sample was 15.22 (SD = 2.25), with 4 missing data points. The distribution of class averages was homogeneous. The largest difference was observed between the first-grade class and the CM2 (last year of primary school) and sixth-grade classes (differences of 3.55 and 2.77, respectively) (Table 1).

Regarding the sample, 83 participants reported having received one or more diagnoses from a medical doctor, psychiatrist, or psychologist (Table 2). The two largest represented categories were “DYS disorders” (dyslexia, dyscalculia, dyspraxia, dysphasia, etc.), which accounted for 11.30% of the participants in relation to the overall sample, and high intellectual potential, which constituted 9.93% of the sample.

The ethics committee of the University of Grenoble-Alpes (CERGA-2022-25) approved this study, and all consents, both from minors and parents, were obtained and validated.

### 2.2. Procedure

The online questionnaire was distributed between June 2022 and January 2023, primarily through social media platforms. Participation was voluntary, and the data collected were anonymous. Several educational institutions assisted in the dissemination, including two public middle schools (169 participants) located in the suburb of Lyon. The questionnaire included two consent forms (one for minors and one for parents) that had to be signed before proceeding to answer the other questions. A lottery reward was offered to participants who completed the entire questionnaire. The three randomly selected participants received €50 each.

Due to the length of the questionnaires for adolescents (estimated at around twenty minutes), the administration of the three questionnaires was randomized to avoid the potential fatigue effect that could lead adolescents to respond more quickly.

### 2.3. Materials

The online questionnaire included consent forms for the minors and adults, as well as questions regarding age, gender, most recent overall grade average, the decision made in the previous year during an educational council (grade repetition, promotion to the next grade, grade skipping, or other), and whether they had received a diagnosis established by a healthcare professional (none, a neurodevelopmental disorder—dyslexia, dysphasia, dyspraxia, attention deficit hyperactivity disorder, autism spectrum disorder—or an affective disorder—depression, generalized anxiety, or other). The adolescents completed three questionnaires, and one questionnaire was filled out by a parent.

Trait anxiety: Assessed using the Revised Children’s Manifest Anxiety Scale (R-CMAS) [50] in its validated French version [51]. Although a more recent French validation by Turgeon and Chartrand [52] was available, it was not chosen due to its Canadian origin and the narrower age range of 8 to 13 years, as compared to the 6-to-19-years age range of the version used in this study. This facilitates its use for similar experiments targeting adolescents and allows for comparisons between different samples. This self-report scale consists of 37 items, and participants indicate “yes” or “no” for each statement. A “yes” response is circled if the child believes the statement is true for them, and “no” is circled if they believe it does not apply to them. The 37 items are divided into three subscales related to anxiety: Physiological anxiety (10 items; e.g., “I wake up scared some of the time”), Worry/Oversensitivity (11 items; e.g., “I worry a lot of the time”), and Social Concerns/Concentration worries (7 items; e.g., “I feel that others do not like the way I do things” or “It is hard for me to keep my mind on my schoolwork”). Additionally, the remaining 9 items constitute the Lie scale, which assesses social desirability or may represent inaccuracies in self-perception. The questionnaire demonstrates good internal consistency for the total anxiety score (α = 0.84) as well as for the Worry/Oversensitivity and Deception scale (0.76), the Social Concerns/Concentration scale (0.69), and to a lesser extent, the Physiological Anxiety scale (0.59). However, the subscale index for physiological anxiety has somewhat lower sensitivity [53].

Emotion regulation strategies: Assessed using the Cognitive Emotion Regulation Questionnaire (CERQ) [30] in its validated French version [54]. The CERQ is a self-report measure that evaluates nine cognitive strategies used to regulate emotions in response to negative or unpleasant events [30]. A total of 36 items are rated on a Likert scale ranging from “almost never” (1) to “almost always” (5) and are distributed among the following nine strategies: Acceptance (A; e.g., “I think that I have to accept the situation”), Positive Refocusing (PRef; e.g., “I think about pleasant experiences”), Refocus on Planning (RP; e.g., “I think about a plan of what I can do best”), Positive Reappraisal (PRea; e.g., “I think I can learn something from the situation”), Putting into Perspective (PP; e.g., “I think that other people go through much worse experiences”), Self-Blame (SB; e.g., “I feel that I am the one to blame for it”), Rumination (R; e.g., “I am preoccupied with what I think and feel about what I have experienced”), Catastrophizing (C; e.g., “I continually think how horrible the situation has been”), and Blaming Others (BO; e.g., “I feel that others are responsible for what has happened”). This questionnaire is suitable for adolescents aged 13 to 19 years. Participants are instructed to reflect on their thoughts when experiencing negative or unpleasant events. An adaptation of this questionnaire has been developed to make it accessible to children aged 10 to 12 years. In the French version, Cronbach’s alphas range from 0.68 to 0.87 for all factors.

Metacognitive beliefs: Assessed using the MetaCognition Questionnaire for Adolescents (MCQ-A) [55] validated in French by Shakeshaft, Lecerf, Morosan, Badoud, and Debbané (MCQ-Af) [56]. Originally developed to support Wells’s metacognitive model of generalized anxiety, this questionnaire has shown its relevance in various emotional disorders. The questionnaire consists of 30 items evenly distributed across five factors: Positive Metacognitive Beliefs (factor MCpos; e.g., “I need to worry in order to work well”), Negative Metacognitive Beliefs (factor MCneg; e.g., “When I start worrying, I cannot stop”), Cognitive Confidence (factor Confidence; e.g., “I have a poor memory”), Negative Beliefs about Thoughts in General linked to superstitions, punishment, and responsibility (factor Control; e.g., “I will be punished for not controlling certain thoughts”), and Cognitive Self-Consciousness (factor Consciousness; e.g., “I monitor my thoughts”). However, it is important to consider items 2 (MCneg factor), 12 (Consciousness), and 14 (Confidence) cautiously, as they presented issues in the French confirmatory analysis. This problem is not new and has been observed in the German sample as well [57]. In this questionnaire, participants rate the statements on a 4-point Likert scale ranging from “Strongly disagree” to “Strongly agree.” For each factor, scores can range from 6 to 24, and the total score ranges from 30 to 120. The MCQ-Af is suitable for adolescents aged 13 to 17 years. An adaptation of this questionnaire has also been developed to make it accessible to children aged 10 to 12 years. In the French version, Cronbach’s alphas range from 0.684 to 0.852 for all five factors and the total score.

Executive functioning: Assessed using the Behavior Rating Inventory of Executive Function (BRIEF) [18,58] in its French version [59]. The BRIEF is a standardized assessment scale designed for parents to provide useful information about a child’s executive functioning in their daily environment. Executive functioning is evaluated based on various behavioral manifestations in children and adolescents aged 5 to 18 years. The questionnaire consists of items that form nine clinical scales [60]: Inhibition (the ability to control impulses and stop one’s own behavior at the appropriate time, including stopping actions and thoughts), Shift (the ability to move freely from one situation to another and think flexibly in order to respond appropriately), Emotional Control (the ability to modulate emotional responses), Initiate (the ability to start activities and generate ideas, answers, and problem-solving strategies), Working Memory (the capacity to hold information in mind for task completion), Plan/Organize (planning: the ability to anticipate events, establish goals, and develop steps for achievement; organization: the ability to evaluate and order key information), Organization of Materials (the ability to impose order on work, play, and storage spaces), Self-monitoring (the ability to monitor the effects of their behavior on others and observe their own behavior in a social context) and Task Monitoring (the ability to check work, evaluating performance during or after task completion to ensure goal attainment). These theoretically and statistically derived scales form two broader indices: the Behavioral Regulation Index (BRI; comprising Inhibition, Shift, and Emotional Control) and the Metacognition Index (MI; comprising Initiate, Working Memory, Plan/Organize, Organization of Materials, and monitoring). A global score (the Global Executive Composite [GEC] index) is also calculable. The questionnaire consists of 86 items, with the last 14 (for the parent form) not used for score calculation (filler items). Parents are asked to evaluate their child’s behavior over the past 6 months using a 3-point Likert scale, indicating whether the behavior has “never,” “sometimes,” or “often” been problematic. These responses are then coded as ordinal variables (1 = never, 2 = sometimes, 3 = often). The BRIEF typically yields eight scale scores and three index scores. In the French version, Cronbach’s alphas range from 0.73 to 0.86 for all factors in the parent form [60].

## 3. Results

### 3.1. Data Exclusion

For the analysis of our data, certain participants were not included, to obtain a more homogeneous sample. Thus, the participant who responded “other” to the gender question was not included in the analyses. The four participants whose decisions did not involve advancing to the next grade were also excluded, as they may not have experienced a similar emotional year compared to the others. Based on the questionnaires, we applied strict eligibility criteria to select participants for inclusion in our analysis. Two participants were excluded due to high scores on the Lie scale of the RCMAS, while two others were retained despite slightly elevated scores on the Negativity scale of the BRIEF. Additionally, ten participants were excluded due to high inconsistency scores on the Inconsistency scale of the BRIEF (for more details, see the Supplementary Materials). These exclusions ensured the reliability and validity of our results, resulting in a final sample that is representative for our analyses. In conclusion, our sample consists of 275 adolescents, including 77 with diagnoses, after implementing these exclusion criteria (17 participants excluded).

### 3.2. Preliminary Analysis 

In accordance with our pre-registration on the Open Science Framework [61], we conducted several analyses to verify if our data behaved as expected based on the literature before testing our hypotheses. Since we primarily used self-reported questionnaires (RCMAS, CERQ, and MCQ), this verification aimed to ensure the reliability of our data. Some of these analyses were not pre-specified, which we have clarified in each case, and we have justified their relevance in the respective sections based on their connection to the literature. Most hypotheses were correlational and did not have a direction. To adhere to the unique model we wanted to test, we used regressions instead of correlations for some analyses. The complete dataset, with supplementary materials, is available on the Mendeley website; see references for access [62].

We conducted several gender difference analyses on our variables, detailed in the Supplementary Materials. All tests controlled for participants’ age and sex. Correlations include confidence intervals based on 1000 bootstrap replicates. In anticipation of the numerous correlation analyses planned in our study, we estimated the sample size using G*Power 3.1.9.7 software. We selected the “exact” family for the statistical test: “Correlation: Bivariate normal model,” and for the type of power analysis, we used “A priori: Compute required sample size.” The alpha risk was set at 0.05, the power at 0.95, and the hypothesized effect size at 0.20. This resulted in a required sample size of 266.

### 3.3. Anxiety

#### 3.3.1. Lying

We conducted this unplanned conformity analysis on the RCMAS Lie scale to determine if our choice to exclude the two participants was justified. A Pearson correlation revealed a significant negative correlation between the Lie subscale and participants’ trait anxiety (*r* = −0.26, *p* < 0.001).

#### 3.3.2. Emotion Regulation Strategies

A linear regression analysis was conducted to determine if ERSs were predicted by trait anxiety. The regression analysis showed that trait anxiety significantly predicted the variance in maladaptive ERSs (*β* = 0.896, *SE* = 0.082, *p* < 0.001, *t* = 10.96, 95% CI [0.73, 1.06]). However, although trait anxiety was negatively associated with adaptive ERSs, the significance threshold was not reached (*p* = 0.062). Thus, the adolescents with a predisposition for experiencing anxiety were more likely to use maladaptive ERSs. A new regression analysis was conducted to distinguish which maladaptive ERSs were likely to be activated based on the adolescents’ trait anxiety. Three out of four maladaptive strategies were significantly and positively predicted by the adolescents’ trait anxiety: Self-Blame (ß = 0.290, *SE* = 0.032, *p* < 0.001, *t* = 10.06, 95% CI [0.23, 0.35]), Rumination (*β* = 0.249, *SE* = 0.037, *p* < 0.001, *t* = 6.801, 95% CI [0.18, 0.32]), and Catastrophizing (*β* = 0.293, *SE* = 0.028, *p* < 0.001, *t* = 9.819, 95% CI [0.23, 0.35]).

#### 3.3.3. Metacognitive Beliefs

This linear regression analysis allowed us to examine the influence of metacognitive beliefs on participants’ total anxiety. It revealed that a high metacognition score (total MCQ) significantly predicted the variance in total anxiety (*β* = 0.303, *SE* = 0.029, *p* < 0.001, *t* = 10.589, 95% CI [0.25, 0.36]). Another linear regression showed that the MCneg factor largely accounted for this prediction (*β* = 0.607, *SE* = 0.084, *p* < 0.001, *t* = 7.222, 95% CI [0.44, 0.77]), followed by the Control factor (*β* = 0.356, *SE* = 0.101, *p* < 0.001, *t* = 3.544, 95% CI [0.16, 0.55]), and Lack of Confidence (*β* = 0.254, *SE* = 0.093, *p* = 0.007, *t* = 2.717, 95% CI [0.07, 0.44]). The MCpos (ß = 0.133, *SE* = 0.093, *t* = 1.433, 95% CI [−0.05, 0.31]) and Consciousness factors (*ß* = 0.022, *SE* = 0.085, *t* = 0.260, 95% CI [−0.14, 0.19]) weakly and non-significantly predicted trait anxiety in a positive direction.

### 3.4. Metacognitive Beliefs and Emotion Regulation Strategies

This analysis was not declared in OSF but provided insights into the relationships between these two variables that have been poorly studied. A Pearson correlation matrix was conducted to determine the strength of the relationships between metacognitive beliefs and ERSs. Maladaptive ERSs showed positive and significant correlations with all subscales of the MCQ (MCpos, *r* = 0.28, 95% CI [0.19, 0.39]; MCneg, *r* = 0.40, 95% CI [0.29, 0.50]; Lack of Confidence, *r* = 0.20, 95% CI [0.08, 0.31]; Control, *r* = 0.42, 95% CI [0.32, 0.51]; and Consciousness, *r* = 0.25, 95% CI [0.14, 0.37], *p* < 0.001). Adaptive ERSs were positively correlated with the MCpos and Consciousness factors. Thus, these subscales were found to be related to both adaptive and maladaptive strategies. The Consciousness subscale was significantly and more strongly correlated with the adaptive ER strategies (*r* = 0.36, *p* < 0.001, 95% CI [0.25, 0.48]) than the MCpos subscale (*r* = 0.11, *p* = 0.065, 95% CI [−0.02, 0.25]). This finding may explain why these two subscales did not predict participants’ anxiety, as they have equally strong associations with both adaptive and maladaptive ERSs.

### 3.5. Executive Functioning and Emotion Regulation Strategies

A Pearson correlation matrix was conducted to explore the relationships between the Global Executive Composite (GEC) index and the various emotion regulation strategies. All the adaptive ERSs were found to be negatively correlated with the GEC index of the BRIEF. Only the Positive Refocusing and Positive Reappraisal strategies showed non-significant associations with EF. Conversely, Acceptance (*r* = −0.15, *p* = 0.013, 95% CI [−0.27, −0.03]), Refocus on Planning (*r* = −0.194, *p* = 0.001, 95% CI [−0.30, −0.08]), and Putting into Perspective (*r* = −0.17, *p* = 0.006, 95% CI [−0.29, −0.05]) showed significant relationships with EF. The maladaptive ERSs were positively correlated with the GEC index. Only the Blaming Others strategy did not show a significant correlation with EF. Self-Blame (*r* = 0.22, *p* < 0.001, 95% CI [0.10, 0.33]), Rumination (*r* = 0.19, *p* < 0.001, 95% CI [0.07, 0.29]), and Catastrophizing (*r* = 0.27, *p* < 0.001, 95% CI [0.16, 0.39]) showed significant relationships with EF.

### 3.6. Analysis of the Relationships between Our Main Variables

We chose to consider our variables of interest through the overall scores of the scales. Indeed, we have so far described the relationships between each of the variables in pairs, to verify the conformity of our data with that of the literature. This allowed us to observe the inconsistency of the MCQ’s Consciousness subscale with emotion regulation strategies and anxiety. We present all analyses (correlation and network analysis) including the Consciousness factor in the Supplementary Materials.

#### 3.6.1. Pearson Correlation

Using a Pearson correlation matrix controlling for age and sex, we found significant associations in our adolescent sample (Table 3). School average was negatively correlated with EF (*r* = −0.36, 95% CI [−0.46, −0.24]), trait anxiety (*r* = −0.18, 95% CI [−0.30, −0.06]), and metacognitive beliefs (*r* = −0.12, 95% CI [−0.26, 0.01]). Trait anxiety positively correlated with maladaptive ERSs (*r* = 0.57, 95% CI [0.48, 0.64]) and metacognitive beliefs (MCQtot, *r* = 0.59, 95% CI [0.50, 0.66]). EFs also showed a positive correlation with trait anxiety (*r* = 0.47, 95% CI [0.36, 0.56]) and metacognitive beliefs (*r* = 0.31, 95% CI [0.19, 0.43]). Maladaptive ERSs correlated with metacognitive beliefs (*r* = 0.49, 95% CI [0.40, 0.58]) and EF difficulties (*r* = 0.27, 95% CI [0.16, 0.38]). Adaptive ERSs correlated negatively with EF (*r* = −0.16, 95% CI [−0.27, −0.05]) and positively with School average (*r* = 0.12, 95% CI [−0.00, 0.25]).

#### 3.6.2. Network Analysis

##### Data Analytic Plan

In accordance with our hypotheses and OSF statement, we initially performed a moderation via mediation analysis, which did not yield significant results (available in the Supplementary Materials). Structural equation models (SEMs) were also conducted, but they all proved to be relevant in various configurations (see [62], JASP file). As suggested by several authors [63,64], we deemed it more judicious and prudent to conduct a network analysis, particularly to maintain the bidirectional rather than unidirectional relationship between anxiety and ERSs. To better understand the link between trait anxiety and executive functioning, we then conducted a network analysis, not originally planned. This approach aimed to develop a comprehensive model integrating emotion regulation strategies and metacognitive beliefs with anxiety and executive functioning. Given the complex interrelationships among our variables and cross-sectional collection, a network analysis was deemed more suitable than a single structural equation model for capturing the dynamics of the relationships between our variables.

##### Network Visualization and Treatment

In accordance with our hypotheses and OSF statement (available in the Supplementary Materials), to conduct our network analysis, we initially relied on rigorously conducted, published articles that used the same procedure, such as that by Heeren and McNally [65]. We employed the JASP software 0.18.1.0 for both analysis and visualization of the network. JASP utilizes the R Bootnet package, following the work of Epskamp et al. [66], and for visualization, it is based on the R qgraph package [67]. Networks are visualized using Fruchterman and Reingold’s [68] algorithm, which organizes nodes according to the strength of their connections, placing nodes with stronger connections near the center and those with weaker connections near the periphery. Solid lines represent positive links, while dotted lines indicate negative correlations. The thicker the line, the stronger the relationship between the two variables. These edges among nodes depict the partial correlations between each pair of variables, allowing for the representation of relationships between two specific variables while eliminating potential influences from other variables [69]. However, the network does not elucidate the causal dynamics among the variables. We selected the EBICglasso Estimator in JASP, which is used to create more interpretable and accurate networks by limiting spurious edges and optimizing the model to best reflect the underlying, real structure of the data [70]. In this function, γ is automatically set to 0.5, favoring caution and reducing the likelihood of obtaining spurious edges.

The JASP software 0.18.1.0 enables the acquisition of network centrality indices. Betweenness centrality quantifies how often a node appears on the shortest paths between pairs of other nodes, signifying its key role as a conduit within the network. Closeness centrality measures the mean distance of a node to all other nodes, showcasing its proximity or accessibility relative to others. Node strength represents the aggregate weight of edges connected to a node, denoting its interaction level. The total weight of incoming edges from other network nodes reflects how much a node is influenced by others, while expected influence is the aggregate weight of outgoing edges from a specific node to others, assessing the extent to which a node affects the network. Higher values for each index suggest greater centrality in the network.

Finally, to increase the stability and accuracy of our network parameters, we employed a non-parametric bootstrapping method with 1000 iterations. The advantage of this technique is that it is exclusively data-driven [66], making it particularly suitable for exploratory work.

##### Standard Network Analysis

**Figure 1:**
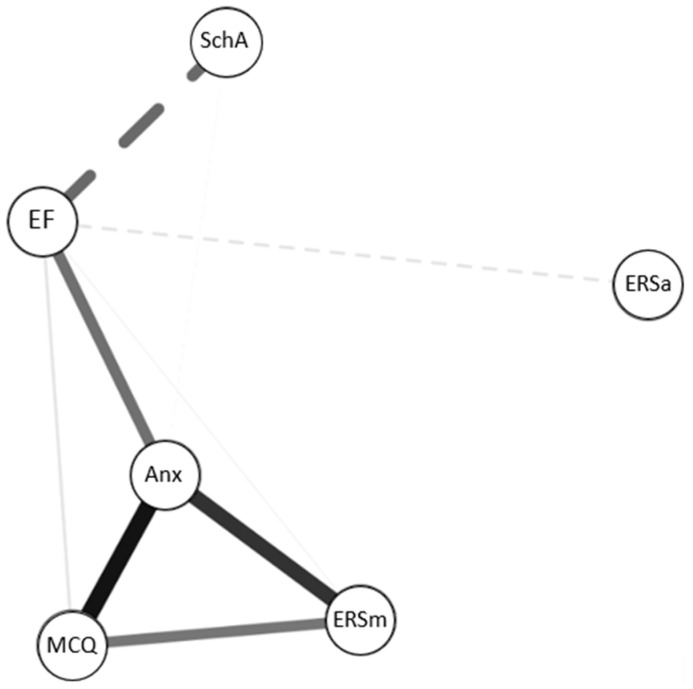
Network analysis of our main variables without the Consciousness factor. Each node represents a variable of interest, and each edge represents the zero-order correlation between two variables. The thickness of an edge reflects the magnitude of the association. Solid lines indicate positive correlations, while dotted lines represent negative correlations. MCQ = MetaCognition Questionnaire without Consciousness factor; ERSm = maladaptive emotion regulation strategies; ERSa = adaptive emotion regulation strategies; EF = executive function; Anx = trait anxiety; SchA = School average.

**Figure 2:**
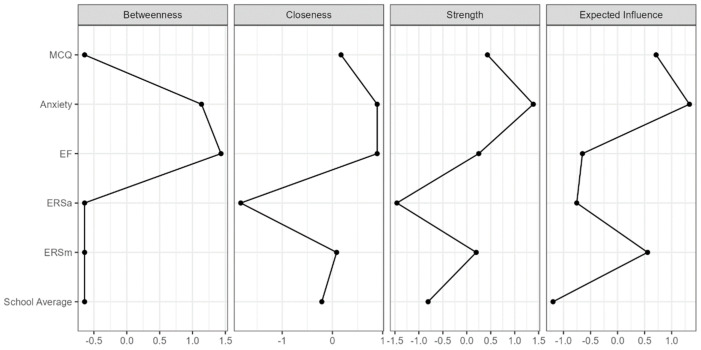
Centrality index graphs. ERSm = maladaptive emotion regulation strategies; ERSa = adaptive emotion regulation strategies; EF = executive function; MCQ = MetaCognition Questionnaire.

We conducted an initial network analysis including all our main variables (see the Supplementary Materials) and then a second network analysis after removing the Consciousness subscale (Figure 1). We have compiled the centrality indices in the Supplementary Materials and illustrated them with graphs (Figure 2).

The characteristics of our network are immediately apparent. Trait anxiety and executive functioning are at the center of our network and have a key role within it. However, strength and expected influence are significantly higher for anxiety than for executive functioning. In contrast, metacognitive beliefs and maladaptive ERSs show more moderate centrality values, suggesting a less central role in the network. The beliefs have higher values compared to ERSm on several indices, suggesting a slightly more influential position in the network. More notably, School average and adaptive ERSs display negative scores on all measures, reflecting a peripheral position and limited influence within the network.

## 4. Discussion

The aim of the current study was to elucidate how emotion regulation strategies and metacognitive beliefs might impact executive functioning and academic achievement in children and adolescents through the mediation of trait anxiety. The examination of these variables was pertinent due to their closely intertwined interconnections. The initial model we had considered did not adequately explain the mechanisms through which emotion regulation strategies and metacognitive beliefs influenced the relationship between trait anxiety and executive functioning (refer to the Supplementary Materials). Rather than further pursuing theoretical predictions using a singular model, we conducted a network analysis of our primary variables. This approach enhanced our understanding of the relationships and provided a robust and broader analysis. It revealed the centrality of trait anxiety in our study. Adolescents who report frequent and varied manifestations of anxiety in their daily lives are likely to struggle in mobilizing functional reflective processes, as represented by metacognitive beliefs and maladaptive emotion regulation strategies. This anxiety is also linked to problematic executive functioning in daily life as reported by parents. Lastly, academic performance was found to be negatively related to executive functioning and, to a lesser extent, adaptive emotion regulation strategies (ERSa). Indeed, ERSa emerged as the most distal, least influential, and least interconnected variable in our network. We discuss the implications of our research by revisiting the comprehensive analyses performed on each variable, comparing these findings to existing literature.

### 4.1. Anxiety 

The various links between our variables and anxiety are generally consistent with the literature. Regarding executive functions (EFs), anxiety was found to be strongly and positively correlated with parent-reported executive functioning problems. While these findings might appear inconsistent with the ACT, it is crucial to note that we did not assess executive functioning performance per se, but rather the behavioral manifestation of executive functioning. The additional cost associated with the use of compensatory strategies to adjust performance in tasks requiring renewed executive functioning in anxious adolescents directly affects their daily lives through their behaviors. Our results indirectly corroborate certain aspects of the ACT. However, we remain cautious, as evaluations based on questionnaires and tasks are conceptually overlapping but provide two different levels of analysis (algorithmic vs. reflective) concerning information processing, as per Stanovich [22,23]. Studies that combine these two levels of analysis could be beneficial to verify and refine the ACT. Although we did not use mediation to explore the links between trait anxiety and academic success, our results suggest that trait anxiety plays a negative role in academic achievement, with EFs also contributing. These findings align with those of Owens et al. [12], who demonstrated a relationship between trait anxiety and academic success mediated by working memory performance in a sample of 50 adolescents with an average age similar to our sample; see also [13,71]. However, these results do not align with those of Alfonso and Lonigan [11]. In their study, trait anxiety was positively linked to EF performance and academic success, with working memory serving as the mediator between trait anxiety and academic outcomes. The authors noted that their sample exhibited low to moderate anxiety scores and that the negative effects of anxiety on performance are more pronounced when stimuli are associated with high threat. Nonetheless, our additional analyses indicated a linear, rather than curvilinear, relationship between anxiety and all our variables, suggesting that anxiety’s impact on adolescent problematic behaviors does not vary with its intensity. As we propose, this discrepancy in results could be attributed to the measure of executive functions.

Regarding emotion regulation strategies (ERSs), the maladaptive ERSs were predicted by trait anxiety in adolescents, except for the Blaming Others strategy. This result is not surprising and has been found in other studies involving both adolescents and adults [72]. This suggests that this strategy may not necessarily be maladaptive, at least in the short term, for reducing the unpleasant effect of an emotion. We note that this strategy is the only one among the ERSs that seeks an external causality for emotional events. Although the relationship between the adaptive ERSs and anxiety did not reach the threshold of significance, this finding is consistent with meta-analyses on the subject, which consistently show weaker associations between adaptive ERSs and anxiety [31,32,33]. Sakakibara and Kitahara [33] provide an interpretation by questioning the reliability of adaptive ERSs and highlighting their lack of conceptual clarity. They suggest revising the items to make them more capable of representing adaptive strategies in the face of unpleasant events. Another approach would be to adapt the questionnaire based on more recent theories by adding or removing strategies. For example, Garnefski et al. [30] used a theoretical approach to create the Cognitive Emotion Regulation Questionnaire (CERQ). This approach Involved building on existing tools by removing, transforming, or adding strategies based on rationality. Therefore, updating the questionnaire is justified in order to enhance its validity and applicability. Incorporating research findings from the field to modify our way of representing, theorizing, or fundamentally conceptualizing emotion regulation in a virtuous cycle could be achieved by drawing on existing models in intervention sciences [73]. Based on current evidence, emotion regulation strategies as assessed through the CERQ do not appear to be efficacious targets for intervention with the aim of diminishing anxiety levels, facilitating the adoption of functional behaviors in adolescents, or enhancing academic performance. In our study, we chose to analyze the influence of anxiety on ERSs, while McLaughlin et al. [36] found the opposite relationship using two separate measurement points. In their study, emotional expression and regulation problems predicted psychopathologies in adolescents, but not vice versa. However, their measures of emotional dysregulation partially differ from our focus on the use of cognitive and conscious strategies to regulate unpleasant emotions. Their questionnaires primarily assessed the understanding of emotions, the regulation of specific emotions (anger and sadness) from a behavioral perspective, and one maladaptive ERS: Rumination. Therefore, caution must be exercised in directly applying their conclusions to our study. Consistent with the ACT model, we believe that the development of cognitive strategies in response to unpleasant emotions depends on one’s state, situation, and psychological traits. Numerous studies have shown that trait anxiety leads to major cognitive biases that make it difficult to disengage the attention from threatening information, e.g., [74]. The cognitive functioning of an anxious person can differ significantly from that of a non-anxious person, e.g., [5,75], suggesting the influence of anxiety on the development of specific strategies.

This indicates that the alignment between a person’s feelings (anxiety) and their actions (ERSs) in response to unpleasant emotions is not always complete and is difficult to superimpose on a single model. As Borkovec [76] suggested, “The reasons an individual generates to explain his or her behavior are often post hoc and unrelated to true causative relationships. But they do provide a view of how chronic worriers and GAD clients perceive their worrying, [...]” (p. 17). In other words, there is a mismatch between one’s feelings (anxiety) and the way they act upon them (ERSs), as the former is based on concrete evidence while the latter attempts to explain the nature of the thoughts driving the regulation strategies.

Regarding the MCQ, the total scale and the subscales of Negative Metacognitive Beliefs (MCneg), Confidence, and Control all predicted anxiety. However, MCneg were significantly more strongly associated with anxiety than the other subscales and the total scale. In contrast, Positive Metacognitive Beliefs (MCpos) and the Consciousness scale did not predict anxiety. These findings may initially seem surprising. However, MCpos and the Consciousness scale consistently show weaker effects on anxiety compared to the other subscales [77,78,79] and may even be absent in certain clinical populations [80]. Conversely, MCneg are more consistently linked to various symptoms, including anxiety, in both clinical and non-clinical populations [49,81,82]. This is consistent with the S-REF model, which highlights the central role of beliefs about uncontrollability and the danger of worry thoughts in maintaining or exacerbating psychological difficulties [8]. Similar results were found for negative metacognitive beliefs in a non-clinical population of 214 French-speaking Swiss adolescents with an average age of 15 years. This study found no influence of the Consciousness variable on anxiety, and the effect of the Lack of Confidence variable became marginally significant (*p* = 0.076) in a stepwise regression. Finally, MCpos were implicated in anxiety. The analysis was based on a version of the MCQ containing 27 items, as three items were found to be inconsistent (items 2, 12, and 14), and four items were included in multiple subscales (items 3, 9, 11, and 23). This version of the scale likely needs refinement by rephrasing these items to improve robustness and reliability. A recent meta-analysis examining healthy adults and adults with psychopathology showed reliable combined effects for all four scales of the MCQ, but the subscale of MCpos was found to be unstable or non-significant [83]. Benedetto et al. [78] proposed that MCpos could be considered an adaptive coping strategy. Our study specifically examined the correlations between ERSs and the MCQ. The correlations of all the MCQ scales were positive and significant with maladaptive strategies. They were stronger for negative beliefs and the need for Thought Control. Positive beliefs are not adaptive, contrary to what Benedetto et al. [78] suggested. Furthermore, the Consciousness scale was also correlated with adaptive strategies, but negatively. This has been observed in healthy subjects [84]. In other words, being conscious of one’s thoughts may be a determining factor in choosing to use an adaptive or maladaptive ERS. This observation reinforces the specificity of the CERQ in measuring conscious cognitive processes, i.e., explicit ERSs [30]. These findings are important to consider when using the MCQ to interpret results in future studies with healthy samples. We hypothesize that the Consciousness subscale might mitigate the outcomes of studies employing the full range of subscales of the MCQ, potentially leading to an increased incidence of Type II errors. Like Sica et al. [84], we believe that rephrasing items in this scale to highlight a more negative aspect of excessive and rigid consciousness could make the questionnaire more discriminating. This confluence of evidence substantiates the rationale for omitting this subscale from our correlation analysis and subsequent network analysis.

### 4.2. Executive Functioning

Our results allowed us to establish a strong link between EFs and academic achievement. Poor executive functioning reported by parents can be a good predictor of an adolescent’s academic success. These findings are consistent with those of Ten Eycke and Dewey [19], who, like us, used the parent version of the BRIEF. They observed correlations between the GEC index and standardized tests of reading and mathematics. Samuels et al. [85] also demonstrated strong correlations, in a four-year longitudinal study of adolescents aged 12 to 15 years, between various school subjects (science, language, mathematics, social studies, etc.) and the GEC index. The fundamental difference is that the assessment of problematic behaviors was conducted by teachers or teaching assistants, not parents. Their correlations were much higher than ours. The lowest correlation was −0.40 (the average correlation between teacher and teacher assistant), and the highest was −0.54, whereas it was −0.35 for our study. Several reasons can explain this difference. Samuels et al. [85] conducted their correlations across multiple subjects, whereas we used the students’ overall averages. It is likely that subjects such as physical education may not necessarily be related to the GEC index or may even have a negative relationship (being beneficial for behaviors). Furthermore, teachers report on adolescents’ behaviors in the classroom, not at home. This assessment is therefore closer to the school reality and more likely to reflect the executive functioning relevant to learning rather than family life. Although weaker, our results suggest that the BRIEF can be considered a relevant tool for assessing EFs in the school environment, whether used by teachers or parents. For a French population, Fournet et al. [60] demonstrated that the BRIEF exhibited greater reliability in its parent-report version compared to the teacher-report version.

Overall, maladaptive emotion regulation strategies (ERSm) were more strongly related to all our variables than adaptive ERSs. They predicted anxiety more strongly, were associated with problematic executive functioning, and also had closer links with metacognitive beliefs than the adaptive ERSs. On the one hand, these results expand those of Lantrip et al. [37] to other ERSs in a similar population and using the same scale for EF assessment (BRIEF). Indeed, they used the Emotion Regulation Questionnaire, which only assesses Reappraisal for adaptive ERSs and Behavioral Suppression for maladaptive ERSs. The CERQ did not allow to assess Behavioral Suppression, but Dramatization, Rumination, and Self-blame were positively correlated with EF. However, although the relationship was also positive, the maladaptive ERS of Blaming Others was not correlated with EF. This finding aligns with the lack of predictive value of anxiety for this subscale and underscores the centrality of anxiety within our network analysis. Furthermore, several adaptive ERSs, such as Putting Things into Perspective, Acceptance, and Focusing on Action, were negatively correlated with problematic EF. On the other hand, these results contradict those of Lantrip et al. [37] regarding Reappraisal, as there was no correlation. One argument could have been that the two types of reappraisal differ conceptually, but strong correlations were found in studies with young adults in American, r = 0.59 [86], and Italian, r = 0.409 [87], samples. The differences are likely due to the characteristics of our sample compared to their study and to the reflective aspect of the ERSs. Our sample size is substantial, and the data have proven to be reliable, which supports the perspective that ERSa as assessed in the CERQ, when viewed from a reflective standpoint, are of limited interest for intervention.

### 4.3. Limitations and Future Directions

One limitation of our study concerns the characteristics of our sample, which may influence the strategies used to regulate emotions, the level of anxiety, and parents’ evaluation of executive functioning. For example, it would be important to have information on the parents’ socioeconomic status as it could directly impact the adolescents’ living conditions. However, the overall academic averages of our sample were high with a moderate standard deviation. This suggests that the parents’ socioeconomic status was relatively high, as several meta-analyses [88,89] have found significant correlations between socioeconomic status and students’ academic performance. Despite this, the lack of such data could have occasionally explained certain behaviors, anxiety levels, strategies employed, problematic behaviors, etc. Indeed, Schäfer et al. [31] suggest that parental support in emotion regulation should play an essential role in a child’s development and provide them with the means to adapt more serenely to daily life. Furthermore, adaptive strategies require a certain cognitive maturity that may be more easily stimulated depending on the parenting style.

Another potential limitation is that, given that we used questionnaires, it is always challenging to draw definite conclusions about the measured concepts and how each question may have been understood and interpreted by the adolescents. We attempted to limit biases by assessing the comprehensibility of the questionnaires and reformulating certain questions (CERQ and MCQ). Although this reformulation remained very close to the original question’s meaning, it is possible that it had an impact on the consistency of the questionnaires or certain scales. As previously highlighted, the CERQ is a questionnaire that focuses on a limited number of strategies that are not all of the same nature. For example, the internal or external causality varies for maladaptive ERSs such as Self-blame and Blaming Others, which can make the scale less consistent. Nevertheless, this questionnaire remains valuable in providing insights into an adolescent’s cognitive style and enabling the development of appropriate interventions in schools or psychological settings. We suggest drawing on more recent work to modify and improve this scale, such as the studies by McRae and Gross [90] and the five families of ERSs. To address these biases, the evaluation of the children’s behavior by parents and the participants’ school averages provided new perspectives and additional sources of information on the students’ behavioral reality to increase objectivity in our data. The fact that our data converged—for example, the self-reported anxiety of the participants was correlated with both the average and the executive functioning reported by the parents—is reassuring regarding the internal validity of our study. These converging data also explain why our correlations rarely exceeded the 0.50 threshold.

The findings of our study on the interplay between trait anxiety, emotion regulation strategies, metacognitive beliefs, and their effects on executive functioning and academic success in adolescents have significant practical implications. Firstly, they advocate for the integration of anxiety management and metacognitive skills development in educational curricula. This could be realized through structured programs or workshops aimed at enhancing students’ self-awareness, emotional regulation abilities, and metacognitive skills. We have focused on the reflexive variables that play a role in anxiety and the mobilization of adolescents’ cognitive abilities for academic success. There are also other reflexive variables like self-efficacy, metacognitive monitoring, and life skills—such as self-awareness and active self-reflection—that are essential in this context. Studies indicate that self-efficacy and metacognitive monitoring significantly influence adolescents’ quality of life and stress perception, in addition to affecting their success in specific areas like mathematics [91,92,93,94,95]. Coupled with ours, targeted interventions focusing on these reflexive variables could be beneficial for both the well-being and success of adolescents. Mindfulness and Acceptance and Commitment Therapies are ideal candidates as they have shown promising effects on these variables, particularly in enhancing emotional regulation, reducing anxiety symptoms, and improving metacognitive awareness and executive functioning [96,97,98,99,100]. Furthermore, our research suggests the need for teacher and parent education on identifying and supporting students struggling with anxiety. This involves training in recognizing signs of high trait anxiety and implementing supportive strategies that encourage positive emotional and cognitive development.

## 5. Conclusions

This study aimed to identify the underlying mechanisms of trait anxiety to explain its detrimental effect on the effectiveness of treatment in mobilizing EFs and achieving academic success. The Attentional Control Theory (ACT) postulates that compensatory strategies employed by individuals with high anxiety allow them to achieve comparable performance but at a higher cognitive cost. Given the literature linking ERSs with trait anxiety and EFs, it was conceivable that these compensatory strategies could be partly represented by ERSs. Although strong links between anxiety, ERSs, and EFs have been independently established, the initial comprehensive model predicting ERSs as a mediator between trait anxiety and EFs, with metacognitive beliefs moderating the link between trait anxiety and ERSs, was not supported. Apparently, ERSm in healthy adolescents are not ideal candidates to represent these compensatory strategies. However, our network analysis highlighted the significance of these reflective variables through the trait anxiety of our participants. These conclusions are based on questionnaire-based evaluations, reflecting a reflective level within Stanovich’s theoretical framework [22,23]. The ACT has been constructed on, and appears to primarily rely on, an algorithmic level, with EF evaluation primarily based on specific tasks. The compensatory strategies mentioned in the ACT may be found at this level rather than the one we assessed. In this regard, ACT theorists [6,101] have proposed that downward attentional networks and reactive attention networks [102], as well as an error correction process called error-related negativity [103], are compensatory mechanisms that could explain the lower treatment effectiveness in individuals with high trait anxiety. They thus appear to be moving towards more objective, tangible, and algorithmic evidence to strengthen their theory. In our study, ERSs and metacognitive beliefs contributed to individual predispositions to experience anxiety. This trait anxiety leads to behavioral issues related to EFs that are the expression of a more costly information processing (algorithmic level). In other words, highly anxious adolescents must exert additional effort to adapt to different situations and mobilize their cognitive resources, which has repercussions on their daily behavior. Thus, we believe that the ACT would benefit from considering this reflective level since, in our view, and in line with Stanovich’s theory, the algorithmic level is dependent on the reflective level. Parallel assessments of these levels, combining performance-based and questionnaire-based evaluations, should be systematically integrated to understand how resources are allocated based on task demands. Measures of executive functions, whether performance-based or questionnaire-based, provide important and non-redundant information about an individual’s effectiveness and success in achieving their goals [21]. Our research shows that the reflective level impacts the behavior of anxious adolescents. It is reasonable to assume that anxious adolescents experience a form of exhaustion that makes it more difficult to mobilize their cognitive capacity for a task and could lead to diminished performance.

## Figures and Tables

**Table 1:** Mean and Distribution of Sample across Educational Levels.

	4th	5th *	6th	7th *	8th **	9th	10th	11th	12th
Mean	15.750	16.500	15.722	15.455	14.627	14.747	14.679	12.950	14.727
SD	1.500	3.162	1.973	1.990	2.611	2.264	2.524	2.712	2.190
*n*	4	5	107	67	32	27	17	12	17

* One missing data ** Two missing data.

**Table 2:** Mean and Distribution of Sample across Diagnoses.

	DYS Troubles	HIP	Anxiety Disorders	Multiple Diagnoses	ADHD	ASD	Total
*n* (%)	33 (11.30)	29 (9.93)	7 (2.39)	10 (3.42)	3 (1.02)	1 (0.34)	83 (100)

HIP = high intellectual potential; ADHD = attention deficit hyperactivity disorder; ASD = autism spectrum disorder; DYS = all neurodevelopmental disorders such as dyslexia, dysphasia, dyspraxia, etc.

**Table 3:** Pearson correlations between main factors and descriptive statistics.

Variable	*n*	*M*	*SD*	1	2	3	4	5	6
1. School average	272	15.26	2.25	—					
2. Trait Anxiety	275	11.24	6.38	−0.184 ***	—				
3. Maladaptive ERSs	275	36.45	9.79	−0.040	0.570 ***	—			
4. Adaptive ERSs	275	53.53	15.67	0.123 *	−0.087	0.083	—		
5. Metacognitive beliefs	275	59.99	11.15	−0.124 *	0.586 ***	0.495 ***	0.016	—	
6. Executive functioning	275	119.85	25.49	−0.359 ***	0.468 ***	0.274 ***	−0.161 **	0.314 ***	—

ERSs = emotion regulation strategies; * *p* < 0.05; ** *p* < 0.01; *** *p* < 0.001.

## Data Availability

The entirety of the data and statistical analysis can be accessed by following this reference: CECILLON, François-Xavier (2023) [62], “Correlational Study in French Adolescents,” Mendeley Data, V4, doi: 10.17632/29mbb556dp.4.

## Supplementary Materials

The following supporting information can be downloaded at https://data.mendeley.com/datasets/29mbb556dp/4. “Correlational Study in French Adolescents,” Mendeley Data, V4, doi: 10.17632/29mbb556dp.4, [62].
